# Risk factors for suicidal attempt in patients with the melancholic subtype of depressive disorder: Implication for nursing care

**DOI:** 10.1097/MD.0000000000029713

**Published:** 2022-08-12

**Authors:** Pengfei Xu, Ying Sun

**Affiliations:** Department of Psychiatry, Tianjin Anding Hospital.

**Keywords:** care, depressive disorder, management, prevention, suicide

## Abstract

The incidence of suicide in patients with depressive order is much higher than other population. We aimed to evaluate the current status and risk of suicidal attempt in patients with the melancholic subtype of depressive disorder, to provide evidence for the clinical management and nursing care of depressive disorder.

Patients diagnosed as the melancholic subtype of depressive disorder and treated in our hospital from June 1, 2018 to August 31, 2021 were included. The characteristics of included patients were collected and analyzed. Pearson correlation analysis and logistic regression analysis with odd ratio and 95% confidence interval were conducted to analyze the influencing factors of suicidal attempt in patients with the melancholic subtype of depressive disorder.

A total of 446 patients with melancholic subtype of depressive disorder were included, the incidence of suicidal attempt was 18.83%. Pearson correlation analysis indicated that gender (*R* = 0.611), alcohol drinking (*R* = 0.719), living situation (*R* = 0.812), number of previous admission to hospital (*R* = 0.547), sleep disorder (*R* = 0.612) and frequent depressive episodes (*R* = 0.559) were all correlated with the suicidal attempt in patients with melancholic subtype of depressive disorder (all *P* < 0.05). Logistic regression analysis showed that female (OR 3.115, 95%CI 2.493–3.906), alcohol drinking(OR 1.946, 95%CI 1.684–2.763), living alone (OR 2.401, 95%CI 1.915–3.008), number of previous admission to hospital ≥ 3 (OR 2.342, 95%CI 1.601–2.742), sleep disorder (OR 1.821, 95%CI 1.328–2.215) and frequent depressive episodes (OR 3.128, 95%CI 2.421–3.779) were the independent risk factors of suicidal attempt (all *P* < 0.05).

Suicidal attempt is common in the patients with melancholic subtype of depressive disorder, and there are many related risk factors for suicidal attempt in patients with the melancholic subtype of depressive disorder.

## 1. Introduction

According to World Health Organization (WHO) statistics, nearly 2 million people commit suicide worldwide in 2020.^[1]^ In China, suicide is the fifth leading cause of death in the general population, and among people aged 15 to 34, suicide is the first cause of death.^[2,3]^ There are many reasons for suicide, and a history of mental illness is a major cause of suicide.^[4]^ Previous studies^[5,6]^ have showed that more than 90% of suicides suffer from at least one mental disorder, and depression patients have the highest suicide rate. The close connection between depression and suicide has been widely recognized by many scholars.

According to the “Diagnostic and Statistical Manual of Mental Disorders, Fourth Edition (DSM-IV)”, depression with melancholic features is a subtype disease unit of depression, accounting for 16%–53% of patients with depressive disorder.^[7,8]^ Compared with other subtypes, depression with melancholic features is more serious, the cognitive function is more impaired, the risk of suicide is higher, and the treatment effect is poor.^[9–11]^ Suicide attempters are high-risk groups of suicide. Therefore, investigating the factors related to suicide attempts can help clinicians identify patients at risk of suicide early and provide early intervention to help reduce the occurrence of suicidal behavior.^[12]^ A study^[13]^ on the suicide risk of depression patients has found that the suicide risk of depression patients with melancholic features was 2.16 times that of those without melancholic features. Therefore, suicide prevention in patients with depression with melancholic characteristics should attract the attention of clinical medical workers.^[14]^ At present, the status and influencing factors of suicide attempts in patients of this subtype remains unclear. Understanding the risk factors for suicidal attempt in patients with the melancholic subtype of depressive disorder is beneficial for health care providers to formulate targeted interventions and nursing care. Therefore, this study targeted on the depression patients with melancholic characteristics, aiming to analyzes the risk factors of suicide attempts in this subtype patients with depressive disorder, to help clinical medical workers identify patients at risk of suicide early, thereby giving targeted intervention and nursing care to reduce the occurrence of suicide.

## 2. Methods

### 2.1. Ethics consideration

In this study, all methods were performed in accordance with the relevant guidelines and regulations. This study was a prospective and cohort design. The study protocol had been checked and verified by the ethical committee of Tianjin Anding Hospital (approval number: CM190021c). Besides, the written informed consents had been obtained from the patients or the guardians of patients.

### 2.2. Patients

Patients diagnosed as the melancholic subtype of depressive disorder and treated in our hospital from June 1, 2018 to August 31, 2021 were included. The criteria of patients’ inclusion were as following: (1) adult patients with age ≥18 years; (2) patients had been diagnosed as melancholic subtype of depressive disorder through the Mini International Neuropsychiatri Interview (MINI).^[15]^ According to MINI, it was defined as accompanied by melancholic features if the answer to either item A5a or A5b was “yes”, and answer “yes” to 3 or more items in A6 section of MINI; (3) patients or related guardians agreed to participant in this study. The patients who did not agree to participant in this study were excluded.

### 2.3. Diagnosis of melancholic subtype of depressive disorder

We defined whether it was accompanied by melancholic features according to the Chinese version of MINI 5.0. A5a: In the last 2 weeks, have you lost interest or pleasure in the things you like on weekdays? A5b: At the time of the most severe depression, do you have no reaction to the things that you liked or that made you feel very happy? If “No,” if something good happens, will it still not make you happy, even for a short time? A6: In the last 2 weeks, when you felt depressed and/or lost interest: a. Is your depression feeling different from the bereavement response? b. Do you feel heavier in the morning almost every day? c. Do you wake up 2 hours earlier than usual almost every morning and cannot fall asleep anymore? d. Does appetite decrease or increase almost every day? Or do not deliberately go on a diet, but lost or gained weight? e. Do you speak or move more slowly almost every day than in the past, or feel irritable, restless, and difficult to sit still? f. Do you feel excessive and unrealistic guilt? A5a or A5b answers “yes,” and 3 or more items in A6 answer “yes,” the patient is judged to be accompanied by melancholic features.

According to the MINI 5.0 Chinese version of the suicide module with good sensitivity and reliability,^[16,17]^ we determined whether the patient had suicidal attempt: C5 Have you ever had an attempted suicide in the last month? C6 Have you ever had an attempted suicide in your life? If you answer “yes” to any of the above questions, it was determined that the patient had attempted suicide.

### 2.4. Data collection

Two psychiatric researchers collected the patient socio-demographic information and clinical characteristics through clinical talks and treatment cases, including age, gender, waist circumference, body mass index, alcohol drinking, smoking, hypertension, diabetes, marry status, working status, education level, living situation, number of previous admission to hospital, sleep disorder, antidepressant use, and frequent depressive episodes. The number of previous hospitalizations refers to the number of hospitalizations due to mental illness; frequent episodes are defined as depressive episodes ≥ 4 times per year; atypical symptoms refer to significant weight gain or increased appetite, excessive sleep, etc; suicidal conception refers to feeling that life is meaningless and hopeful I have died or I often think about death-related things or negative thoughts, number of previous admission to hospital, sleep disorder, antidepressant use, and frequent depressive episodes. The number of previous admission to hospital refers to the number of hospitalizations due to mental illness; frequent depressive episodes were defined as depressive episodes ≥ 4 times per year.

### 2.5. Statistical methods

In this study, SPSS 23.00 software was used for statistical analysis of data. The distribtution of data was preexamined before statistical analysis. Persistent variables were expressed as mean ± standard deviation, comparison between groups is by *t* test; categorical variables were expressed as percentage (%), and comparison between groups was performed by chi-square test. Taking suicide attempt as the dependent variable, variables with statistically significant differences between groups were included in the multivariate logistic regression with odd ratio and 95% confidence interval to analyze the risk factors of suicide attempt in patients with depression with melancholic characteristics. Pearson correlation analysis was conducted to analyze the correlation of suicidal attempt and related characteristics. In this study, *P* < 0.05 was considered that the difference between the groups was statistically significant.

## 3. Results

A total of 446 patients with melancholic subtype of depressive disorder were included in this present study, of whom 84 patients had the suicidal attempt, the incidence of suicidal attempt in patients with melancholic subtype of depressive disorder was 18.83%. As indicated in Table 1, there were significant differences in the gender, alcohol drinking, living situation, number of previous admission to hospital, sleep disorder and frequent depressive episodes between suicidal attempt group and no- suicidal attempt group (all *P* < 0.05). There were no significant differences in the age, waist circumference, body mass index, smoking, hypertension, diabetes, marry status, working status, education level and antidepressant use between suicidal attempt group and no- suicidal attempt group (all *P* > 0.05).

**Table 1:** The characteristics of patients with depressive disorder suicidal attempt in patients with depressive disorder.

Variables	Suicidal attempt group (n = 84)	No- suicidal attempt group (n = 362)	t/χ^2^	*P*
Age (y)	41.06 ± 7.33	40.69 ± 8.17	7.022	0.085
Male/female	12/72	125/237	1.458	0.021
Waist circumference (cm)	82.61 ± 23.46	81.44 ± 21.92	5.017	0.089
BMI (kg/m^2^)	23.03 ± 2.93	22.95 ± 3.15	3.043	0.071
Alcohol drinking	69(82.14%)	164(45.30%)	2.392	0.015
Smoking	10(11.90%)	42(11.60%)	1.955	0.114
Hypertension	55(65.48%)	232(64.08%)	3.013	0.059
Diabetes	29(34.52%)	120(33.15%)	1.925	0.101
Marital status			1.116	0.063
Unmarried	16(19.05%)	68(18.78%)		
Married	68(80.95%)	250(69.07%)		
Divorced	12(14.29%)	44(12.15%)		
Working status			2.091	0.077
Employed	39(46.43%)	176(%)		
Unemployed	45(53.57%)	186(51.38%)		
Education level			1.144	0.095
Illiteracy	10(11.90%)	40(11.05%)		
Primary school	22(26.19%)	91(25.14%)		
Junior school	35(41.67%)	166(45.86%)		
Senior school	11(13.09%)	36(9.94%)		
College	6(7.14%)	29(8.01%)		
Living situation			2.059	0.041
Alone	58(69.05%)	50(13.81%)		
Living with spouse/partner	16(19.05%)	185(51.10%)		
Living with children	5(5.95%)	20(5.53%)		
Pension agency	3(3.57%)	103(28.45%)		
other	2(2.38%)	4(1.11%)		
Number of previous admission to hospital	4.03 ± 1.81	1.66 ± 0.97	1.606	0.012
Sleep disorder	71(84.52%)	175(48.34%)	2.089	0.011
Antidepressant use	65(77.38%)	280(77.35%)	1.271	0.106
Frequent depressive episodes	77(91.67%)	201(55.52%)	3.174	0.038

### 3.1. Pearson correlation analysis

As showed in Table 2, Pearson correlation analysis indicated that gender (*R* = 0.611), alcohol drinking (*R* = 0.719), living situation (*R* = 0.812), number of previous admission to hospital (*R* = 0.547), sleep disorder (*R* = 0.612) and frequent depressive episodes (*R* = 0.559) were all correlated with the suicidal attempt in patients with melancholic subtype of depressive disorder (all *P* < 0.05).

**Table 2:** Pearson correlation analysis of suicidal attempt and related characteristics.

Variables	*R*	*P*
Age (y)	0.201	0.068
Gender	0.611	0.025
Waist circumference (cm)	0.204	0.073
BMI (kg/m^2^)	0.197	0.104
Alcohol drinking	0.719	0.041
Smoking	0.055	0.103
Hypertension	0.124	0.078
Diabetes	0.192	0.089
Marry status	0.205	0.114
Working status	0.104	0.042
Education level	0.136	0.088
Living situation	0.812	0.004
Number of previous admission to hospital	0.547	0.048
Sleep disorder	0.612	0.028
Antidepressant use	0.154	0.112
Frequent depressive episodes	0.559	0.027

### 3.2. Logistic regression analysis

The variable assignments of multivariate logistic regression were presented in Table 3. As presented in Table 4, Logistic regression analysis showed that female (OR 3.115, 95%CI 2.493–3.906), alcohol drinking (OR 1.946, 95%CI 1.684–2.763), living alone (OR 2.401, 95%CI 1.915–3.008), number of previous admission to hospital ≥ 3 (OR 2.342, 95%CI 1.601–2.742), sleep disorder (OR 1.821, 95%CI 1.328–2.215) and frequent depressive episodes (OR 3.128, 95%CI 2.421–3.779) were the independent risk factors of suicidal attempt (all *P* < 0.05).

**Table 3:** The variable assignments of multivariate logistic regression.

Factors	Variables	Assignment
Suicidal attempt	Y	Yes = 1, no = 2
Gender	X_1_	Female = 1, male = 2
Alcohol drinking	X_2_	Yes = 1, no = 2
Living situation	X_3_	Alone = 1, not alone = 2
Number of previous admission to hospital	X_4_	≥3 = 1, <3 = 2
Sleep disorder	X_5_	Yes = 1, no = 2
Frequent depressive episodes	X_6_	Yes = 1, no = 2

**Table 4:** Logistic regression analysis on the risk factors of suicidal attempt.

Variables	β	Wald	OR	95%CI	*P*
Female	0.121	0.148	3.115	2.493–3.906	0.016
Alcohol drinking	0.115	0.181	1.946	1.684–2.763	0.032
Living alone	0.134	0.156	2.401	1.915–3.008	0.029
Number of previous admission to hospital≥3	0.141	0.178	2.342	1.601–2.742	0.015
Sleep disorder	0.127	0.165	1.821	1.328–2.215	0.041
Frequent depressive episodes	0.106	0.234	3.128	2.421–3.779	0.011

## 4. Discussions

The results of this study have indicated that among depression patients with melancholic features, 18.83% of patients have attempted suicide, and female, alcohol drinking, living alone, number of previous admission to hospital ≥ 3, sleep disorder and frequent depressive episodes are the independent risk factors of suicidal attempt, early targeted intervention and nursing care are needed for those patients. Suicide brings incalculable losses to individuals, family, and society. It is not only a serious social problem, but also an important public health problem that is increasingly concerned in the world of mental health research. Suicide is a complex social behavior, which may be determined by multiple factors such as genetics and social psychology.^[18]^ Mental disorders are an important cause of suicide, among which the suicidal behavior of patients with depression is the most common.

There are gender differences in the occurrence of depression, and this difference appears from adolescence.^[19]^ Gender differences in depression will inevitably lead to differences in suicidal behavior among depression patients.^[20]^ The intensity of suicidal ideation in women with depression is higher than that of men, and the number of suicidal behaviors in the past is more than that of men.^[21]^ Previous study^[22]^ has found that 69% of female depressed patients have said that they had suicidal thoughts, while 53% of male depressed patients have said that they had thought of suicide. However, studies^[23,24]^have also found that men have a higher incidence of suicidal ideation than women. In terms of the possession of social resources, men have more opportunities and more resources than women.^[25]^ The level of education leads to uneven employment opportunities, which further affects women’s social and economic status.^[26]^ In social life, the incidence of unmarried, divorced, or widowed female patients is significantly higher than that of male patients, which suggests that female patients may face more marital and emotional problems.^[27]^ This point can be found in the statistical results of negative life events, women pay more attention to negative events in marriage and emotion, while men may focus more on work and career.

For patients living alone, they may have higher risks of suicidal attempt. Good social support is conducive to health, while the existence of bad social relations harms physical and mental health.^[28]^ Social support provides protection to individuals under stress and plays a role in buffering stress. Besides, good social support is important to maintain a good emotional experience.^[29]^ It is reported that patients with depression lack a complete social support system. When they are depressive, they cannot get outside support well or cannot make effective use of support.^[30,31]^ The results of previous studies^[32–34]^ have shown that depression patients with low levels of social support increase the risk of suicide, which is more consistent with the results of this study. Therefore, for female patients with depression who live alone, attention should be paid to giving care and special attention.

Alcohol abuse and alcohol dependence are very common phenomena in China, and related problems caused by drinking are increasing rapidly.^[35]^ Previous studies^[36–38]^ have shown that drinking problems are one of the important reasons for suicides in rural China, and the prevalence of alcohol dependence among suicide attempters is high. Studies^[39,40]^ have used psychological anatomy to study the association between community environment and suicide behavior, and they have found that there is a statistically significant difference between alcoholism in the case group and the control group, and the prevalence of alcoholism among suicide deaths is higher than that in the control group. In addition, many studies^[26,41]^ have shown that alcoholics have a higher risk of suicide, and dangerous drinking is an independent risk factor for suicide. People with depression may seek comfort by drinking alcohol. Therefore, patients with depression should give up alcohol as soon as possible and provide necessary social and psychological support.

Sleep disorder is one of the common symptoms of depression, including difficulty falling asleep, waking up easily, waking up early, and lack of sleep perception, among which early waking is the most obvious characteristic.^[42]^ Previous studies^[43,44]^ have shown that sleep disorders may aggravate patients’ depression and suicidal thoughts. More and more studies^[45,46]^ have shown that sleep disturbance is an independent risk factor for suicidal ideation, suicide attempted behavior, and suicide death. The results of many previous studies^[47,48]^ have shown that improving patients’ sleep can help improve patients’ positive attitude and improve their quality of life. Therefore, for patients with depression, medical workers should actively take care of their sleep state and take necessary nursing measures to improve the patient sleep in order to reduce the occurrence of suicidal tendencies.

The results of previous studies^[49,50]^ suggest that the number of previous hospitalizations and frequent depressive episodes are related to the suicide attempts of patients. The results of this research also support this conclusion. Social support is an important protective factor for the formation of suicidal ideation, and good social support is a good buffer system for stress events.^[51]^ Effective social support can prevent the suicidal behavior of patients with depression and eliminate or reduce suicidal ideation.^[52]^ Previous studies^[53,54]^ have also showed that positive coping styles can help regulate the severity of suicidal ideation. On the contrary, poor response methods will expand the negative cognition of depression patients, further aggravate the degree of depression, and lead to suicidal behavior.^[55,56]^ Therefore, for patients with depression, especially female patients, continuously improving their personality characteristics, giving more psychological support and a relaxed living environment, may reduce the risk of suicide and reduce the occurrence of suicide.^[57,58]^

There are certain limitations in this study that are worth considering. First of all, although this study is a prospective research design, there are some chronic diseases other than hypertension and diabetes associated with a higher risk of depression, we collect as much personal and treatment information related to patients as possible, but we still fail to collect and analyze other factors that may be related to suicide attempts, such as comorbid personality disorder, substance dependence, etc. Secondly, the population of this study are all from inpatients of our hospital, and there is a lack of community samples; Thirdly, some variables analyzed in this study may have recall bias. Finally, the results of this study can only provide a certain basis for the relationship between the suicide risk of depressed patients and the characteristics of related patients, and cannot form a causal inference. It needs to be further analyzed and discussed in the future with large samples in different regions.

## 5. Conclusions

In summary, the results of this study have indicated that the incidence of suicidal attempt in patients with the melancholic subtype of depressive disorder is 18.83%, and for patients with female, alcohol drinking, living alone, number of previous admission to hospital ≥ 3, sleep disorder and frequent depressive episodes, they may have higher risk of suicidal attempt.

## Author contributions

Y S designed research; P X, Y S conducted research; Y S analyzed data; Y S wrote the first draft of manuscript; P X, Y S had primary responsibility for final content. All authors read and approved the final manuscript.
